# Single-session radiofrequency ablation for the treatment of benign cystic thyroid nodules: A prospective study

**DOI:** 10.1371/journal.pone.0341556

**Published:** 2026-02-04

**Authors:** Van Bang Nguyen, Trong Binh Le, Chi Le Van

**Affiliations:** 1 Center of Endocrinology and Diabetes, Family Hospital, Da Nang, Vietnam; 2 Department of Internal Medicine, Hue University of Medicine and Pharmacy, Hue University, Hue City, Vietnam; 3 Department of Radiology, Hue University of Medicine and Pharmacy, Hue University, Hue City, Vietnam; Universidad de Las Americas, Quito-Ecuador, ECUADOR

## Abstract

**Background:**

Benign cystic thyroid nodules are commonly encountered in clinical practice and often require intervention due to compressive symptoms or cosmetic concerns. While ethanol ablation (EA) has been widely used, radiofrequency ablation (RFA) has emerged as a promising alternative, particularly for nodules with a significant solid component. This study aimed to evaluate the short-term efficacy and safety of single-session RFA in treating purely cystic and predominantly cystic thyroid nodules (PCTNs).

**Methods:**

This prospective study included 38 patients with benign cystic thyroid nodules (10 purely cystic and 28 PCTNs) treated with single-session RFA. Pre- and post-treatment evaluations were performed using ultrasound imaging and thyroid function tests at 1, 6, and 12 months. The primary endpoint was volume reduction rate (VRR), and therapeutic success was defined as VRR > 50%. Complications were assessed according to international image-guided tumor ablation criteria. This study was registered at ClinicalTrials.gov (identifier: NCT07115576).

**Results:**

RFA resulted in significant volume reduction, with VRR improving from 65.97 ± 22.19% at 1 month to 83.29 ± 17.33% at 6 months and 82.49 ± 23.18% at 12 months (p = 0.004). Purely cystic nodules demonstrated higher VRR than PCTNs at all follow-up points (p < 0.05). Thyroid function remained stable post-procedure. The therapeutic success rate was 83.3% at 1 month and 86.7% at 12 months, with no major complications reported. Purely cystic nodules showed a consistently higher and more durable response than PCTNs.

**Conclusion:**

Single-session RFA appears to be a safe and effective minimally invasive treatment option for large benign thyroid nodules, although confirmation in comparative studies is needed. The technique is particularly advantageous for purely cystic nodules and offers a minimally invasive alternative to surgery or multiple EA sessions.

**Trial registration:**

ClinicalTrials.gov NCT07115576

## Introduction

Thyroid nodules are common in adults and, in population-based cohorts assessed with high-frequency ultrasonography, their prevalence has been reported to range from 20% to 76% [1,2]. In Vietnam, the overall prevalence of thyroid nodules was 48.4%, significantly higher in women than in men (55.2% vs. 42.9%; p < 0.001), with advanced age, hypertension, hyperglycemia, and metabolic abnormalities identified as associated factors [3]. Partially cystic nodules comprise 53.5% of all thyroid nodules, with 13.7% characterized as predominantly cystic, containing more than 75% cystic components [4]. Management of benign cystic thyroid nodules primarily depends on the severity of compressive symptoms or cosmetic concerns [5].

Radiofrequency ablation (RFA) has been demonstrated to be an effective treatment for cystic thyroid nodules, with outcomes influenced by factors such as solid proportion, vascularity, and nodule size [6]. However, the optimal treatment technique for thyroid cystic nodules remains a topic of debate. For purely cystic thyroid nodules, RFA has shown a significantly greater volume reduction within the first three months post-treatment, but this advantage diminishes over longer follow-ups, with similar outcomes observed at six months [6]. For predominantly cystic thyroid nodules (PCTNs), RFA has been associated with a higher volume reduction rate (VRR) and a lower recurrence risk compared to other techniques such as simple aspiration and ethanol ablation (EA), likely due to its ability to address the vascularity and solid components contributing to regrowth or bleeding [7,8]. In a randomized controlled trial, Baek et al*.* reported that RFA achieved a mean VRR of 87.5 ± 11.5%, compared with 82.4 ± 28.6% obtained with EA after six months [9].

Despite promising results, studies directly comparing the short-term efficacy and safety of single-session RFA for both purely cystic nodules and PCTNs remain limited, particularly in Vietnam. This study aims to evaluate the one-month, 6 months, 12 months outcomes of single-session RFA for these types of nodules.

## Methods

### Study design and patient’s selection

This prospective study was conducted in accordance with the Declaration of Helsinki and approved by the Ethics Committee of the Institutional Review Board of University of Medicine and Pharmacy, Hue university, Hue, Vietnam (Number: H2023/050). Also, this analysis is part of the SS-RFAT study (ClinicalTrials.gov Identifier: NCT07115576). Written informed consent was obtained from all participants prior to their inclusion in the study.

From June 2023 to April 2025, patients who underwent thyroid RFA for cystic thyroid nodules at the Centre of Endocrinology and Diabetes, Danang Family Hospital. Patients were included if they met the following criteria: [1] thyroid nodules with a largest diameter ≥ 20 mm [2] nodules with greater than 50% cystic composition; [3] presence of symptoms or cosmetic concerns; [4] documented thyroid function, including serum free thyroxine (FT4) and thyrotropin (TSH) levels; [5] cytology-confirmed benign thyroid nodules through one or two ultrasound-guided FNA procedures; [6] refusal of surgical treatment; and [7] ability to complete at least a one-month, 6 months and 12 months follow-up post-ablation. The exclusion criteria were as follows: [1] nodules with a largest diameter of less than 20 mm; [2] current thyrotoxicosis; [3] a short life expectancy due to severe comorbid diseases; [4] pregnancy; and [5] loss to follow-up.

Before the procedures, all patients were provided with a detailed explanation of the RFA technique, including its potential benefits and risks, to ensure informed decision-making.

### Pre-ablation assessment

All patients underwent ultrasound examinations conducted by a single experienced radiologist at the same hospital. Ultrasound-guided fine-needle aspiration (FNA) procedures were performed by an endocrinologist (Nguyen VB), who holds a professional certification and has over five years of experience in thyroid interventions. A real-time ultrasound system (Acuson NX2 or NX3, Siemens Medical Solutions, California, USA) equipped with an 8–12 MHz linear probe was used for all cases. Ultrasound evaluation included measurement of nodule size and assessment of internal fluid content. Three diameters of each thyroid nodule were recorded, and the volume was calculated using the formula: V = πabc/6, where *V* is the volume and *a*, *b*, and *c* represent the three measured diameters. Nodules were classified as purely cystic if the solid component accounted for less than 10% of the total volume, and as PCTNs if the solid component ranged from 10% to 50% [10]. Thyroid function tests were obtained prior to the procedure and at 1, 6, and 12 months post-treatment.

### Procedure

The RFA procedure was conducted in an outpatient setting with patients positioned in the supine position and the neck slightly extended. The entire procedure was performed under ultrasound guidance by a single endocrinologist with over five years of experience and certified to perform this technique. Following skin sterilization and local anesthesia with 2% lidocaine at the needle insertion site and the peri-thyroidal region, an 18-gauge needle was used to puncture the thyroid cyst under ultrasound guidance via the trans-isthmic approach. The internal cystic fluid was aspirated as completely as possible using a 20 ml or 50 ml syringe. In cases where the cyst contained highly viscous colloid material, a larger-bore 16-gauge needle was employed for aspiration, followed by saline injection to facilitate the removal of colloid before ablation [11].

Before ablating the thyroid nodule and its vascular structures, the cystic fluid was entirely removed. A cooled, internally irrigated monopolar electrode (with a 5 mm or 7 mm active tip) connected to a radiofrequency generator (CoATherm AK-F200, APRO KOREA Inc., Gyeonggi-do, Korea) was then introduced into the nodule using the trans-isthmic approach under ultrasound guidance. The moving-shot technique was applied to systematically ablate the nodule unit by unit. Hydro-dissection was performed by slowly injecting 5% dextrose solution to protect critical structures such as nerves and arteries. Additionally, Doppler ultrasonography was used to identify and target vascular structures within and around the nodule to ensure thorough ablation. Complete ablation of the nodule was confirmed by the appearance of a transient hyperechoic zone on ultrasound imaging, indicating successful treatment [11–13].

### Follow-up

Follow-up assessments, including ultrasound imaging, thyroid function tests, and clinical examinations, were conducted at one month, six months, and 12 months post-procedure, using the same evaluation methods as those performed before RFA, allowing assessment of both early treatment effects and mid-term nodule changes [14]. Treatment efficacy, defined as the primary endpoint, was determined by measuring the VRR, with therapeutic success classified as VRR > 50% at each follow-up US examination [15]. Safety outcomes, considered as the secondary endpoint, were evaluated based on the guidelines established by the international working group on image-guided tumor ablation. Major complications were defined as those leading to significant morbidity or disability, requiring an increased level of medical care, hospital admission, or blood transfusion due to severe bleeding, as well as cases resulting in permanent voice changes. Minor complications included transient side effects such as pain or temporary voice alterations.[16].


$$\begin{array}{c} \text{VRR}\;(\%)\;=\;\frac{\left(\text{Baseline}\;\text{volume--}\text{1}\;\text{month}\;\text{posttreatment}\;\text{volume}\right)}{\text{Baseline}\;\text{volume}}\;\text{X}\;\text{100}\% \end{array}$$

### Statistical analysis

Statistical analyses were conducted using SPSS version 20.0 for Windows. The sample size was estimated based on a previous study reporting a mean VRR of 87.5 ± 11.5% for RFA treating cystic thyroid nodules [9]. Using a 95% confidence level (α = 0.05) and a permissible error (E) of 4%, the minimum required sample size was calculated as 32 participants, according to the formula:


$$\text{n}=Z_{\left(\text{1}-\frac{\alpha }{\text{2}}\right)\;}^{\text{2}}\text{x}\;\frac{\sigma^{\text{2}}}{d^{\text{2}}}$$

- n represents the minimum required sample size
- Z(1−α/2) is the standard normal deviate corresponding to a 95% confidence level
- σ is the standard deviation of the VRR obtained from a previous study (11.5%)
- d is the permissible error, set at 4%, which corresponds to the margin of error around the estimated mean VRR.

An additional 10% was added to account for potential loss to follow-up, resulting in a final target sample size of 36 patients. In this study, sample size is 38 patients.

Safety outcomes were presented as the number of events along with their respective percentages. To evaluate RFA efficacy, the mean and standard deviation (SD) of the volume reduction ratio (VRR) at 1 month, 6 months and 12 months post-ablation were calculated. Changes in nodule volume, largest diameter, FT4, TSH, VRR, and therapeutic success rates from baseline to 1, 6, and 12 months post-treatment were assessed using a Linear Mixed Model, with fixed effects being follow-up time and nodule characteristics. Multiple linear regression analysis was used to identify independent predictors of efficacy, specifically VRR at 6 months. Statistical significance was defined as a p-value of less than 0.05.

## Results

**Fig 1:**
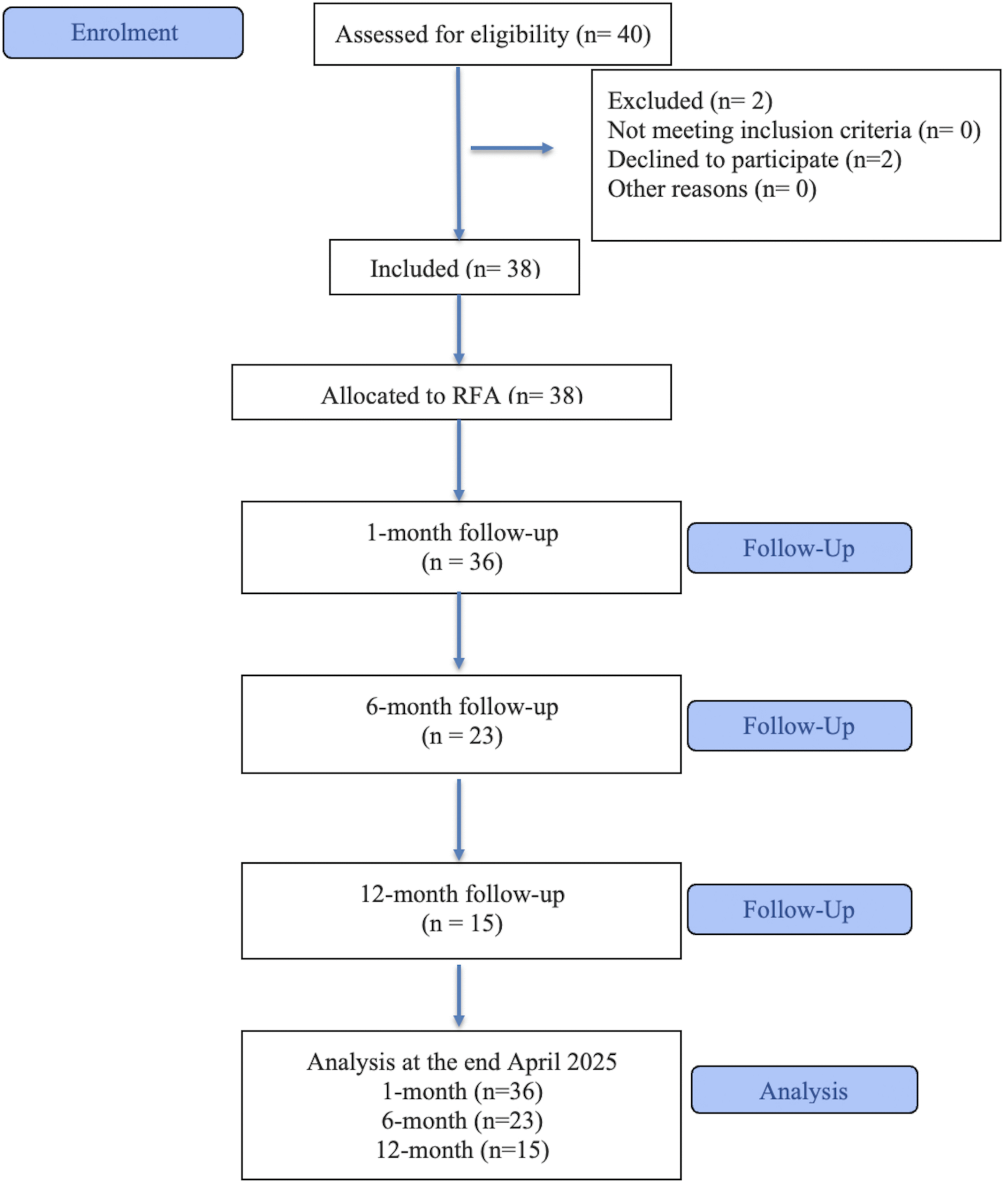
Consort flow diagram of patient enrollment and follow-up.

From June 2023 to April 2025, a total of 38 patients diagnosed with either a purely thyroid cyst or PCTNs were deemed eligible for inclusion through the Ehealth program. Specifically, 36 patients completed the 1-month follow-up, 23 continued to the 6-month follow-up, and 15 remained under observation at the 12-month mark at the end April 2025. Patients lost to follow-up did not return for scheduled visits due to reasons such as relocation, inability to be contacted, or personal scheduling constraints; none of the losses were related to procedure-associated adverse events. The flow of participants through the study is shown in Fig 1. The study was conducted without any complications, either major or minor.

Table 1 shows baseline characteristics of 38 patients. A total of 38 patients were enrolled in the study, including 4 males (10.5%) and 34 females (89.5%), with a mean age of 41.6 ± 18.5 years. The mean largest nodule diameter was 22.8 ± 10.0 mm (range, 10–37.6 mm), and the average nodule volume was 10.1 ± 9.1 mL (range, 0.84–36.96 mL). The mean TSH and FT4 concentrations were 1.37 ± 0.93 mIU/L (range, 0.11–4.77 mIU/L) and 1.22 ± 0.18 ng/dL (range, 0.76–1.52 ng/dL), respectively. Purely thyroid cysts accounted for 26.3% of cases, while 73.7% were PCTNs. The mean aspirated fluid volume was 6.0 ± 7.1 mL (range, 1–35 mL). Regarding anatomical distribution, 53.3% of nodules were located in the right lobe, 42.1% in the left lobe, and 2.6% in the isthmus. The mean baseline cosmetic and symptom scores were 3.00 ± 0.82 and 4.42 ± 0.71, respectively. The procedure duration averaged 16.2 ± 3.1 minutes, with lidocaine used for local anesthesia. The mean energy delivered was 16.72 ± 1.66 kJ, while the minimum and maximum RF powers were 22.5 ± 4.15 watts and 43.15 ± 11.17 watts, respectively.

**Table 1:** Baseline characteristics of 38 patients and nodules.

Characteristic	Summary statistics (N = 38)
Gender (n, %)	
Male	4 (10.5)
Female	34 (89.5)
Age (year), (mean ± SD) (range)	41.6 ± 15.9 (14-77)
Largest diameter (mm), (mean ± SD) (range)	32.0 ± 8.6 (20-57.0)
Nodule volume (mL), (mean ± SD) (range)	10.1 ± 9.1 (1.41-36.96)
TSH (microUI/ml), (mean ± SD) (range)	1.37 ± 0.93 (0.11-4.17)
FT4 (ng/dl), (mean ± SD) (range)	1.22 ± 0.18 (0.76-1.52)
Type of thyroid cyst (n,%)	
Purely thyroid cysts	10 (26.3)
PCTNs	28 (73.7)
Volume of fluid aspirated (ml) (mean ± SD) (range)	6.0 ± 7.1 (1-35)
Location (n,%)	
Right lobe	21 (55.3)
Ismuth	1 (2.6)
Left lobe	16 (42.1)
Cosmetic score (mean ± SD) (range)	3.02 ± 0.98 (1 –4 )
Symptom score (mean ± SD) (range)	4.02 ± 4.17 (0-10)
Time of procedure (mean ± SD) (range)	12.64 ± 9.31 (2-42)
Vlidocain (mean ± SD) (range)	8.42 ± 3.5 (4 –20 )
Energy (mean ± SD) (range)	16.72 ± 16.66 (1.37-70)
Power min(mean ± SD) (range)	22.5 ± 4.15 (20 –30 )
Power max (mean ± SD) (range)	43.15 ± 11.87 (25-70)

TSH, Thyrotropin; FT4, Free Thyroxine; PCTNs, predominantly cystic thyroid nodules; SD, Standard deviation.

Table 2 presents the clinical outcomes observed following the initial session of RFA therapy. Following RFA, both the largest diameter and volume of the thyroid nodules showed a significant reduction over time. The mean largest diameter decreased from 32.00 ± 8.55 mm at baseline to 16.21 ± 9.27 mm at 12 months (p < 0.0001), and the mean nodule volume was reduced from 10.14 ± 9.05 mL to 2.04 ± 3.95 mL over the same period (p = 0.0001). These changes indicate a marked and sustained volumetric response to RFA treatment. In contrast, thyroid function tests, including serum TSH and FT4 levels, remained relatively stable throughout the follow-up. The TSH concentration changed slightly from 1.37 ± 0.93 to 1.45 ± 1.01 μIU/mL (p = 0.673), and FT4 levels showed no significant variation (from 1.22 ± 0.18 to 1.24 ± 0.22 ng/dL, p = 0.610)

**Table 2:** Treatment outcomes of initial radiofrequency ablation session.

Characteristic	Treatment outcomes				p
	Initial (N = 38)	1 month (N = 36)	6 months (N = 23)	12 months (N = 15)	
**Largest diameter (mm), (mean ± SD)**	32.00 ± 8.55	22.17 ± 7.02	16.25 ± 8.02	16.21 ± 9.27	0.0001
**Nodule volume (mL), (mean ± SD)**	10.14 ± 9.05	3.13 ± 3.07	1.52 ± 1.94	2.02 ± 3.95	0.0001
**TSH (microUI/ml), (mean ± SD)**	1.37 ± 0.93	1.24 ± 0.83	1.55 ± 0.96	1.45 ± 1.01	0.673
**FT4 (ng/dl), (mean ± SD)**	1.22 ± 0.18	1.26 ± 0.20	1.19 ± 0.15	1.24 ± 0.22	0.61

(*), p-value for both the Ethanol Ablation and Radiofrequency Ablation Groups.

TSH, Thyrotropin; FT4, Free Thyroxine; SD, Standard deviation.

Table 3 summarizes the outcomes of RFA for thyroid cyst treatment at 1-month, 6-month, and 12-month follow-up intervals. The overall VRR after RFA showed a statistically significant improvement over time, increasing from 65.97 ± 22.19% at 1 month to 83.29 ± 17.33% at 6 months and slightly maintaining at 82.49 ± 23.18% at 12 months (p = 0.001). When stratified by nodule type, purely thyroid cysts exhibited a higher VRR at all time points compared to PCTNs, although the difference was not statistically significant (p = 0.45). Specifically, purely cystic nodules reached a VRR of 77.58 ± 17.04% at 1 month and 91.12 ± 10.86% at 6 months, maintaining a high reduction of 86.87 ± 13.04% at 12 months (p < 0.05). In contrast, the VRR for PCTNs increased from 61.50 ± 22.59% at 1 month to 81.64 ± 8.19% at 6 months and 81.82 ± 24.68% at 12 months.

**Table 3:** Follow-up outcomes of radiofrequency ablation for thyroid cysts at 1, 6, and 12 months.

Outcome	Treatment outcomes			p
	1 month (N = 36)	6 months (N = 23)	12 months (N = 15)	
**VRR (%)**	65.97 ± 22.19	83.29 ± 17.33	82.49 ± 23.18	0.001
**VRR in Purely thyroid cyst**	77.58 ± 17.04	91.12 ± 10.86	86.87 ± 13.04	0.45
**VRR in PCTN**	61.50 ± 22.59	81.64 ± 8.19	81.82 ± 24.68	
**Therapeutic success rate (n, %)**	30/36 (83.33)	22/23 (95.65)	13/15 (86.67)	0.365
**Therapeutic success rate in Purely thyroid cyst**	9/10 (90.0)	4/4 (100)	2/2 (100)	0.96
**Therapeutic success rate in PCTN**	21/26 (80.77)	18/19 (94.73)	11/13 (84.61)	0.33

VRR, Volume Reduction Rate; TSH, Thyrotropin; FT4, Free Thyroxine; PCTNs, predominantly cystic thyroid nodules; SD, Standard deviation.

Regarding treatment efficacy, the overall therapeutic success rate declined over time from 30/36 cases at 1 month to 13/15 at 12 months, with a no statistically significant difference (p = 0.365). Notably, the therapeutic success rate remained consistent and high in purely cystic nodules (from 9/10 at 1 month to 2/2 at 12 months, p > 0.05), whereas in PCTNs, it decreased from 21/26–11/13 over the same timeframe.

According to the multiple linear regression analysis, no significant correlations were observed between the volume reduction ratio (VRR) at one and six months after ablation and any of the evaluated clinical or ultrasonographic parameters, including demographic data, thyroid function indicators, procedural details, and specific nodule features

## Discussion

In recent years, RFA have been one of the minimally invasive alternative treatments of benign thyroid nodule(s) (including solid-cystic nonfunctioning benign nodules, autonomously functioning thyroid nodules), low-risk papillary thyroid carcinoma, and recurrent thyroid cancers [17–24].

Our prospective study conducted from June 2023 to April 2025 evaluated the efficacy and safety of single-session RFA for treating benign cystic thyroid nodules, encompassing both purely cystic nodules (less than 10% solid component) and predominantly cystic thyroid nodules (PCTNs, 10–50% solid component). In our study the mean nodule diameter and volume significantly decreased from 32.0 mm to 16.2 mm and from 10.1 mL to 2.0 mL at 12 months (p < 0.001). The overall VRR improved from 66.0 ± 22.2% at 1 month to 83.3 ± 17.3% at 6 months and 82.5 ± 23.2% at 12 months. Purely cystic nodules achieved higher VRR (up to 91.1% at 6 months) compared with predominantly cystic nodules (81.6%). The therapeutic success rate was 83.3% at 1 month and 86.7% at 12 months, and no major complications or thyroid function changes were observed. Our findings demonstrated that RFA is a safe and effective minimally invasive treatment, achieving significant VRR without major complications or impact on thyroid function.

Our findings are consistent with prior research on RFA for benign thyroid nodules. A randomized controlled trial by Ha et al. (2015) [9] compared RFA and EA for PCTNs and found no significant difference in VRR at 6 months (87.5% ± 11.5% for RFA vs. 82.4% ± 28.6% for EA, p = 0.710). The VRR in our cohort at 1 month was 65.97%. While this is a promising result, comparisons with findings from other studies that differ in design and patient populations cannot be used to infer relative efficacy. This short-term advantage could be attributed to RFA’s ability to target vascular structures and ablate solid components more effectively, as facilitated by the moving-shot technique and Doppler ultrasound used in our study.

Jeong et al. (2022) [25] evaluated the impact of RFA and EA on thyroid-specific quality of life (QoL) in patients with symptomatic benign thyroid nodules. Both treatments significantly improved QoL, but EA demonstrated higher VRR for nodules with a higher fluid component (solidity <50%) at 1, 6, and 12 months (78.7% ± 16.1%, 86.3% ± 21.7%, and 90.9% ± 14.9%, respectively) compared to RFA for nodules with solidity ≥50% (49.1% ± 15.8%, 73.0% ± 14.5%, and 80.3% ± 12.4%). These findings support our observation that purely cystic nodules respond better to RFA than PCTNs, which contain a higher solid component requiring more extensive ablation.

Recurrence remains a significant concern with EA. Sung et al. (2015) [26] reported a recurrence rate of 38.3% after EA for predominantly cystic thyroid nodules, with initial nodule volume >20 mL and high vascularity (grade >1) as independent predictors of recurrence. In contrast, our study reported no recurrences during the 12-month follow-up, although longer-term data are needed to confirm this. The absence of recurrence in our cohort may be due to the targeted ablation of vascular structures using Doppler ultrasound and the moving-shot technique, which enhances the precision and efficacy of RFA.

Lee et al. (2010) (8) proposed RFA as a supplementary treatment for nodules with incomplete resolution after EA, particularly those with residual solid components. This is particularly relevant for PCTNs, where solid portions may persist post-EA, leading to recurrence. Our study’s success with PCTNs, although with lower VRR than purely cystic nodules, supports the role of RFA in managing more complex nodules.

The superior efficacy of RFA for purely cystic nodules can be attributed to the complete removal of cystic fluid followed by ablation of the nodule wall, which prevents fluid reaccumulation. For PCTNs, the presence of solid components necessitates more extensive ablation, potentially explaining the lower VRR compared to purely cystic nodules. The use of the moving-shot technique and Doppler ultrasound to target vascular structures likely enhanced the efficacy of RFA by reducing the risk of incomplete ablation and subsequent recurrence.

Compared to EA, RFA’s ability to ablate both cystic and solid components makes it more versatile, particularly for nodules with higher solid content or those at risk of recurrence. However, EA’s simplicity and lower cost make it a more accessible option in resource-limited settings. The choice between RFA and EA should therefore consider nodule characteristics, patient preferences, and economic factors.

### Limitations

Our study has several limitations that warrant consideration. First, the follow-up period of 12 months, while sufficient to assess short-term efficacy, may not be adequate to evaluate long-term outcomes, such as recurrence rates. Second, the sample size of 38 patients is relatively small, limiting the generalizability of the findings. Third, the study design incorporated some retrospective elements, which may introduce selection bias. Fourth, due to incomplete medical records, data on vascularity, cosmetic scores, and symptom scores were unavailable, precluding a comprehensive assessment of treatment outcomes beyond VRR. Finally, the lack of a direct comparison group receiving EA within the same study population limits our ability to definitively conclude RFA’s superiority.

### Clinical implications

RFA offers a safe and effective minimally invasive alternative to surgery for patients with benign cystic thyroid nodules, particularly those who refuse surgery or have contraindications. The high VRR and absence of major complications make RFA an attractive option, especially for purely cystic nodules. For PCTNs, RFA remains effective but may require careful patient selection due to lower VRR compared to purely cystic nodules. Implementing RFA more broadly in clinical practice could help optimize resource allocation-particularly in low- and middle-income countries such as Vietnam, where access to surgical care may be limited.

### Future research directions

To address the limitations of this study, future research should focus on larger, multicenter, randomized controlled trials with extended follow-up periods to compare RFA and EA comprehensively. These studies should evaluate not only VRR but also patient-centered outcomes, such as quality of life, cosmetic scores, and symptom relief. Cost-effectiveness analyses are also critical, particularly in low-resource settings where EA’s lower cost may influence treatment decisions. Additionally, exploring the potential of combining RFA and EA or investigating other ablation techniques, such as laser or microwave ablation, could optimize treatment strategies for complex nodules. Developing clinical guidelines based on nodule characteristics and patient profiles would further standardize treatment selection.

## Conclusion

In conclusion, single-session RFA appears to be a safe and effective minimally invasive treatment option for benign cystic thyroid nodules. Further research is needed to refine treatment algorithms and enhance patient outcomes in the management of benign cystic thyroid nodules.

## Supporting information

S1 File Supplementary Information for the Database Study. (XLSX)

S2 File TREND checklist. (DOCX)

## Abbreviations

EA: Ethanol ablation
FNA: Fine Needle Aspiration
FT4: Free Thyroxine
PCTNs: predominantly cystic thyroid nodules
RF: Radiofrequency
RFA: Radiofrequency ablation
SD: Standard deviation
TSH: Thyrotropin
VRR: Volume Reduction Rate
US: ultrasound
