# Supplementary material for: Single-session radiofrequency ablation for the treatment of benign cystic thyroid nodules: A prospective study
Source: PLoS One. 2026 Feb 4;21(2):e0341556. doi: 10.1371/journal.pone.0341556 (PMC12872029; doi:10.1371/journal.pone.0341556)
Supplement: S2 File — (DOCX) [file pone.0341556.s002.docx]

**TREND Checklist for "Single-Session Radiofrequency Ablation for the Treatment of Benign Cystic Thyroid Nodules: A Prospective Study": ClinicalTrials.gov identifier: NCT07115576**

| **Item No.** | **Descriptor** | **Reported?** | **Details and Comments** |
| --- | --- | --- | --- |
| **Title and Abstract** |  |  |  |
| 1 | **Identification as a study of a behavioral or public health intervention using a nonrandomized design** | Yes | The title clearly identifies the study as evaluating "Single-Session Radiofrequency Ablation" (an intervention) for thyroid nodules, and the prospective design implies nonrandomized. The abstract summarizes the intervention, methods, and outcomes. |
| 2 | **Structured abstract summarizing key elements of the study** | Yes | The abstract includes Background, Methods, Results, and Conclusion, summarizing the study’s purpose, methods, key findings (VRR, safety), and implications. |
| **Introduction** |  |  |  |
| 3 | **Scientific background and explanation of rationale** | Yes | The introduction provides background on benign cystic thyroid nodules, their clinical significance, and the rationale for evaluating RFA as an alternative to ethanol ablation (EA) or surgery. It cites relevant literature to support the study’s purpose. |
| 4 | **Specific objectives or hypotheses** | Yes | The objective is clearly stated: to evaluate the short-term efficacy and safety of single-session RFA for purely cystic and predominantly cystic thyroid nodules (PCTNs). No specific hypotheses are stated, but this is acceptable for an exploratory study. |
| **Methods** |  |  |  |
| 5 | **Description of intervention(s), including how and when administered** | Yes | The Methods section details the RFA procedure (e.g., trans-isthmic approach, moving-shot technique, hydro-dissection, ultrasound guidance) and follow-up assessments at 1, 6, and 12 months. Timing and administration are clear. |
| 6 | **Description of comparison group(s), if applicable** | No | The study lacks a comparison group (e.g., EA or control). Historical comparisons with EA are discussed in the Discussion, but no direct comparison group is included in the study design. **Suggestion**: Consider adding a comparison group or explicitly justifying the single-arm design. |
| 7 | **Eligibility criteria for participants** | Yes | Inclusion criteria are clearly listed: nodules >50% cystic, symptomatic or cosmetic concerns, benign cytology via FNA, refusal of surgery, and ability to follow up. |
| 8 | **Settings and locations where data were collected** | Yes | Data were collected at the Centre of Endocrinology and Diabetes, Danang Family Hospital, Vietnam, from June 2023 to April 2025. |
| 9 | **Method of participant selection and recruitment** | Partial | The manuscript mentions patients were enrolled via the Ehealth program, but details on recruitment (e.g., how patients were identified or approached) are limited. **Suggestion**: Provide more details on recruitment methods (e.g., referrals, advertisements). |
| 10 | **Sample size, including rationale for the number of participants** | Partial | The study includes 38 patients, but no rationale or power calculation is provided to justify the sample size. **Suggestion**: Include a justification for the sample size, even if based on feasibility or exploratory aims. |
| 11 | **Assignment method for allocating participants to interventions** | N/A | As a single-arm study, no allocation method is needed. All participants received RFA. |
| 12 | **Unit of analysis (e.g., individual, group, community)** | Yes | The unit of analysis is individual patients, clearly indicated by per-patient outcomes (VRR, thyroid function). |
| 13 | **Outcomes, including primary and secondary outcomes, and how they were measured** | Yes | Primary outcome: Volume reduction rate (VRR), measured via ultrasound using the formula V = πabc/6, with therapeutic success defined as VRR >50%. Secondary outcome: Safety, assessed per international image-guided tumor ablation criteria. Thyroid function (TSH, FT4) was also measured. |
| 14 | **Statistical methods used to compare groups or assess outcomes** | Yes | Statistical methods include repeated measures ANOVA or Friedman test for changes in outcomes, Wilcoxon signed-rank test for non-normal data, and multiple linear regression to identify predictors of VRR. Methods are appropriate for a single-arm study. |
| **Results** |  |  |  |
| 15 | **Participant flow, including numbers at each stage and reasons for loss to follow-up** | Yes | A CONSORT flow diagram (Figure 1) shows 40 eligible patients, 2 declining RFA, 38 treated, and follow-up numbers (36 at 1 month, 23 at 6 months, 15 at 12 months). Reasons for loss to follow-up are not specified. **Suggestion**: Clarify reasons for dropout at each follow-up stage. |
| 16 | **Dates defining the periods of recruitment and follow-up** | Yes | Recruitment and treatment occurred from June 2023 to April 2025, with follow-up at 1, 6, and 12 months post-procedure. |
| 17 | **Baseline characteristics of participants** | Yes | Table 1 provides detailed baseline characteristics (e.g., age, gender, nodule size, TSH, FT4, nodule type, location, cosmetic/symptom scores). |
| 18 | **Outcomes and estimation, including measures of effect and variability** | Yes | Tables 2 and 3 report outcomes (VRR, largest diameter, nodule volume, TSH, FT4) with means, standard deviations, and p-values. Therapeutic success rates are reported with percentages. |
| 19 | **Ancillary analyses, if any** | Yes | Multiple linear regression analysis was conducted to explore predictors of VRR, finding no significant correlations. |
| **Discussion** |  |  |  |
| 20 | ****Interpretation of Interpretation of the results, taking into account study hypotheses, sources of potential bias, imprecision of measures, multiplicative analyses, and other limitations or weaknesses of the study** | Yes | The Discussion interprets findings in the context of prior studies, compares RFA with EA, and acknowledges limitations (e.g., small sample size, short follow-up, lack of comparison group). It discusses clinical implications and future research directions. |
| 21 | **Generalizability of findings** | Yes | The manuscript acknowledges the small sample size as a limitation to generalizability but does not explicitly discuss the population to which findings might apply. |
| 22 | **Overall evidence and implications for practice** | Yes | The Conclusion and Discussion highlight RFA as a safe, effective alternative to surgery, particularly for purely cystic nodules, and call for further research to refine treatment algorithms. Clinical implications and future research directions are well-articulated |
